# 3-Bromo-5-*tert*-butyl-2-hy­droxy­benz­alde­hyde

**DOI:** 10.1107/S1600536811048847

**Published:** 2011-11-19

**Authors:** V. Balasubramani, T. Vinuchakkaravarthy, Sreeraj Gopi, S. Narasimhan, D. Velmurugan

**Affiliations:** a Asthagiri Herbal Research Foundation, 162-A, Industrial Estate, Perungudi, Chennai 600 092, India; b Centre of Advanced Study in Crystallography and Biophysics, University of Madras, Maraimalai Campus (Guindy Campus), Chennai 600 025, India

## Abstract

The mol­ecular conformation of the title compound, C_11_H_13_BrO_2_, is stabilized by an intra­molecular O—H⋯O hydrogen bond. All non-H atoms except the methyl groups lie approximately in a common plane (r.m.s. deviation = 0.011 Å).

## Related literature

For the biological activity of substituted salicyl­aldehyde and its derivatives, see: Mounika *et al.* (2010 ▶); Dueke-Eze *et al.* (2010 ▶); Jesmin *et al.* (2010 ▶). For a related structure, see: Wang *et al.* (2010 ▶).
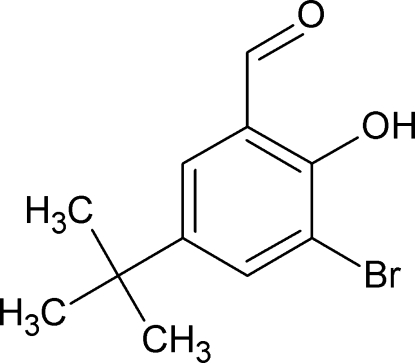


## Experimental

### 

#### Crystal data

C_11_H_13_BrO_2_
*M* *_r_* = 257.11
Orthorhombic, 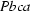

*a* = 9.9727 (19) Å
*b* = 12.174 (2) Å
*c* = 18.558 (3) Å
*V* = 2253.0 (7) Å^3^
*Z* = 8
Mo *K*α radiation
μ = 3.62 mm^−1^
*T* = 293 K
0.2 × 0.2 × 0.2 mm

#### Data collection

Bruker SMART APEXII area-detector diffractometer
11555 measured reflections
2808 independent reflections
1442 reflections with *I* > 2σ(*I*)
*R*_int_ = 0.079

#### Refinement

*R*[*F* ^2^ > 2σ(*F* ^2^)] = 0.056
*wR*(*F* ^2^) = 0.162
*S* = 1.02
2808 reflections
131 parameters
H-atom parameters constrained
Δρ_max_ = 0.59 e Å^−3^
Δρ_min_ = −0.49 e Å^−3^

### 

Data collection: *APEX2* (Bruker, 2008 ▶); cell refinement: *SAINT* (Bruker, 2008 ▶); data reduction: *SAINT*; program(s) used to solve structure: *SHELXS97* (Sheldrick, 2008 ▶); program(s) used to refine structure: *SHELXL97* (Sheldrick, 2008 ▶); molecular graphics: *ORTEP-3* (Farrugia, 1997 ▶); software used to prepare material for publication: *SHELXL97* and *PLATON* (Spek, 2009 ▶).

## Supplementary Material

Crystal structure: contains datablock(s) global, I. DOI: 10.1107/S1600536811048847/bt5688sup1.cif

Structure factors: contains datablock(s) I. DOI: 10.1107/S1600536811048847/bt5688Isup2.hkl

Supplementary material file. DOI: 10.1107/S1600536811048847/bt5688Isup3.cml

Additional supplementary materials:  crystallographic information; 3D view; checkCIF report

## Figures and Tables

**Table 1:** Hydrogen-bond geometry (Å, °)

*D*—H⋯*A*	*D*—H	H⋯*A*	*D*⋯*A*	*D*—H⋯*A*
O2—H2*A*⋯O1	0.82	1.93	2.650 (6)	145

**Fig. 1.**
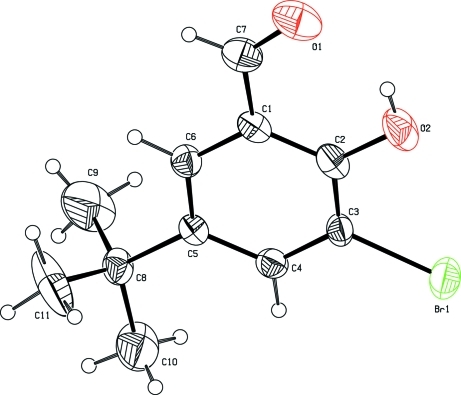
The molecular structure of the title compound, showing displacement ellipsoids drawn at the 30% probability level. H atoms are presented as a small spheres of arbitrary radius.

## supplementary crystallographic information

### Comment

The crystal structure determination of the title compound was undertaken as a part of the synthesis, structure and properties of new substituted salicylaldehyde derivatives.

In the title compound, the substituted aldehyde group, hydroxy group and and bromine are essentially coplanar with the benzene ring with a plane mean deviation of 0.025 (6)°, -0.029 (4)°, -0.015 (1)° and -0.015 (1)°, respectively. An intramolecular O2—H2A···O(1) hydrogen bonding observed between the oxygen atoms of the hydroxy group and the aldehyde group stabilizes the molecular structure.

### Experimental

The synthesis of the title compound follows the modified Riemmer-Tiemann reaction, in which the substituted salicylaldehydes were synthesized from substituted phenols. To 80 mL of water 60g of sodium hydroxide was added and dissolved completely. Then 15g of 4-*tert*-butyl phenol was added and heated to 60-65°C. 30 mL chloroform was added step by step to the mixture. The resulting reaction mixture was heated for one hour, until the formation of precipitate. The liquid layer containing 5-*tert*-butyl-2-hydroxy benzaldehyde as the product was separated through suction pump. It was then brominated using liquid bromine and acetic acid. The final product 3-bromo-5-*tert*-butyl-2-hydroxy benzaldehyde with a maximum yield of 83% was checked for purity using TLC.

### Refinement

Hydrogen atoms were placed in calculated positions with O—H = 0.82Å, C_aromatic_—H = 0.93Å, C_methyl_—H = 0.96Å and refined using a riding model with fixed isotropic displacement parameters *U*_iso_(H) = 1.5 *U*_eq_(C_methyl_, O) or *U*_iso_(H) = 1.2 *U*_eq_(C_aromatic_) or.

### Crystal data

**Table:**

C_11_H_13_BrO_2_	*F*(000) = 1040
*M**_r_* = 257.11	*D*_x_ = 1.516 Mg m^−^^3^
Orthorhombic, *P**b**c**a*	Mo *K*α radiation, λ = 0.71073 Å
Hall symbol: -P 2ac 2ab	Cell parameters from 2808 reflections
*a* = 9.9727 (19) Å	θ = 2.2–28.3°
*b* = 12.174 (2) Å	µ = 3.62 mm^−^^1^
*c* = 18.558 (3) Å	*T* = 293 K
*V* = 2253.0 (7) Å^3^	Block, red
*Z* = 8	0.2 × 0.2 × 0.2 mm

### Data collection

**Table:**

Bruker SMART APEXII area-detector diffractometer	1442 reflections with *I* > 2σ(*I*)
Radiation source: fine-focus sealed tube	*R*_int_ = 0.079
graphite	θ_max_ = 28.3°, θ_min_ = 2.2°
ω and φ scans	*h* = −13→13
11555 measured reflections	*k* = −16→16
2808 independent reflections	*l* = −24→24

### Refinement

**Table:**

Refinement on *F*^2^	Secondary atom site location: difference Fourier map
Least-squares matrix: full	Hydrogen site location: inferred from neighbouring sites
*R*[*F*^2^ > 2σ(*F*^2^)] = 0.056	H-atom parameters constrained
*wR*(*F*^2^) = 0.162	*w* = 1/[σ^2^(*F*_o_^2^) + (0.0685*P*)^2^ + 1.3566*P*] where *P* = (*F*_o_^2^ + 2*F*_c_^2^)/3
*S* = 1.02	(Δ/σ)_max_ < 0.001
2808 reflections	Δρ_max_ = 0.59 e Å^−^^3^
131 parameters	Δρ_min_ = −0.49 e Å^−^^3^
0 restraints	Extinction correction: *SHELXL97* (Sheldrick, 2008), Fc^*^=kFc[1+0.001xFc^2^λ^3^/sin(2θ)]^-1/4^
Primary atom site location: structure-invariant direct methods	Extinction coefficient: 0.0228 (17)

### Special details

**Table:**

Geometry. All esds (except the esd in the dihedral angle between two l.s. planes) are estimated using the full covariance matrix. The cell esds are taken into account individually in the estimation of esds in distances, angles and torsion angles; correlations between esds in cell parameters are only used when they are defined by crystal symmetry. An approximate (isotropic) treatment of cell esds is used for estimating esds involving l.s. planes.
Refinement. Refinement of F^2^ against ALL reflections. The weighted R-factor wR and goodness of fit S are based on F^2^, conventional R-factors R are based on F, with F set to zero for negative F^2^. The threshold expression of F^2^ > 2sigma(F^2^) is used only for calculating R-factors(gt) etc. and is not relevant to the choice of reflections for refinement. R-factors based on F^2^ are statistically about twice as large as those based on F, and R- factors based on ALL data will be even larger.

### Fractional atomic coordinates and isotropic or equivalent isotropic displacement parameters (Å^2^)

**Table:**

	*x*	*y*	*z*	*U*_iso_*/*U*_eq_	
C1	0.2770 (5)	0.1032 (4)	0.2246 (2)	0.0474 (11)	
C2	0.3680 (5)	0.0161 (4)	0.2178 (2)	0.0507 (12)	
C3	0.4296 (4)	−0.0219 (3)	0.2794 (2)	0.0454 (10)	
C4	0.4025 (4)	0.0242 (3)	0.3456 (2)	0.0462 (10)	
H4	0.4468	−0.0027	0.3860	0.055*	
C5	0.3112 (4)	0.1097 (3)	0.3540 (2)	0.0422 (10)	
C6	0.2493 (5)	0.1482 (3)	0.2924 (2)	0.0473 (11)	
H2	0.1878	0.2054	0.2960	0.057*	
C7	0.2094 (6)	0.1487 (4)	0.1612 (3)	0.0656 (14)	
H7	0.1509	0.2073	0.1680	0.079*	
C8	0.2802 (5)	0.1613 (4)	0.4275 (2)	0.0526 (12)	
C9	0.1367 (8)	0.1334 (7)	0.4478 (4)	0.136 (3)	
H9A	0.1272	0.1358	0.4992	0.204*	
H9B	0.1153	0.0610	0.4308	0.204*	
H9C	0.0770	0.1857	0.4262	0.204*	
C10	0.3682 (8)	0.1149 (6)	0.4877 (3)	0.104 (2)	
H10A	0.4604	0.1319	0.4780	0.156*	
H10B	0.3570	0.0367	0.4901	0.156*	
H10C	0.3423	0.1471	0.5328	0.156*	
C11	0.3014 (10)	0.2826 (5)	0.4242 (3)	0.131 (4)	
H11A	0.3899	0.2977	0.4063	0.196*	
H11B	0.2919	0.3133	0.4716	0.196*	
H11C	0.2362	0.3149	0.3926	0.196*	
O1	0.2253 (5)	0.1147 (3)	0.1006 (2)	0.0919 (14)	
O2	0.3953 (4)	−0.0316 (3)	0.15377 (18)	0.0749 (10)	
H2A	0.3523	−0.0009	0.1219	0.112*	
Br1	0.55350 (5)	−0.13980 (4)	0.27226 (4)	0.0729 (3)	

### Atomic displacement parameters (Å^2^)

**Table:**

	*U*^11^	*U*^22^	*U*^33^	*U*^12^	*U*^13^	*U*^23^
C1	0.050 (3)	0.052 (2)	0.040 (2)	−0.012 (2)	0.001 (2)	−0.0048 (19)
C2	0.050 (3)	0.056 (2)	0.047 (3)	−0.017 (2)	0.013 (2)	−0.012 (2)
C3	0.041 (2)	0.0376 (19)	0.057 (3)	−0.0019 (17)	0.005 (2)	−0.0125 (18)
C4	0.046 (2)	0.045 (2)	0.047 (3)	−0.004 (2)	−0.005 (2)	−0.0026 (19)
C5	0.047 (3)	0.042 (2)	0.038 (2)	−0.0035 (18)	0.001 (2)	−0.0045 (16)
C6	0.052 (3)	0.043 (2)	0.047 (3)	0.001 (2)	0.000 (2)	−0.0012 (17)
C7	0.073 (4)	0.076 (3)	0.048 (3)	−0.003 (3)	−0.010 (3)	0.002 (2)
C8	0.065 (3)	0.057 (3)	0.036 (2)	0.010 (2)	0.007 (3)	−0.0029 (19)
C9	0.095 (5)	0.231 (10)	0.081 (5)	−0.019 (6)	0.037 (5)	−0.059 (5)
C10	0.142 (7)	0.119 (5)	0.052 (3)	0.040 (5)	−0.007 (4)	−0.004 (3)
C11	0.276 (12)	0.059 (3)	0.057 (3)	0.006 (5)	−0.001 (5)	−0.017 (3)
O1	0.111 (4)	0.119 (3)	0.046 (2)	−0.009 (3)	−0.015 (2)	−0.001 (2)
O2	0.080 (2)	0.092 (2)	0.053 (2)	−0.007 (2)	0.019 (2)	−0.0289 (18)
Br1	0.0566 (4)	0.0588 (4)	0.1033 (6)	0.0078 (2)	0.0097 (3)	−0.0204 (3)

### Geometric parameters (Å, °)

**Table:**

C1—C6	1.400 (6)	C8—C11	1.494 (7)
C1—C2	1.401 (7)	C8—C9	1.518 (9)
C1—C7	1.465 (7)	C8—C10	1.528 (8)
C2—O2	1.350 (5)	C9—H9A	0.9600
C2—C3	1.377 (6)	C9—H9B	0.9600
C3—C4	1.377 (6)	C9—H9C	0.9600
C3—Br1	1.899 (4)	C10—H10A	0.9600
C4—C5	1.392 (6)	C10—H10B	0.9600
C4—H4	0.9300	C10—H10C	0.9600
C5—C6	1.381 (6)	C11—H11A	0.9600
C5—C8	1.533 (6)	C11—H11B	0.9600
C6—H2	0.9300	C11—H11C	0.9600
C7—O1	1.209 (6)	O2—H2A	0.8200
C7—H7	0.9300		
			
C6—C1—C2	120.3 (4)	C9—C8—C10	106.1 (5)
C6—C1—C7	118.9 (4)	C11—C8—C5	109.8 (4)
C2—C1—C7	120.8 (4)	C9—C8—C5	108.6 (5)
O2—C2—C3	119.8 (4)	C10—C8—C5	112.6 (4)
O2—C2—C1	122.3 (4)	C8—C9—H9A	109.5
C3—C2—C1	117.9 (4)	C8—C9—H9B	109.5
C4—C3—C2	121.1 (4)	H9A—C9—H9B	109.5
C4—C3—Br1	119.8 (4)	C8—C9—H9C	109.5
C2—C3—Br1	119.1 (3)	H9A—C9—H9C	109.5
C3—C4—C5	122.2 (4)	H9B—C9—H9C	109.5
C3—C4—H4	118.9	C8—C10—H10A	109.5
C5—C4—H4	118.9	C8—C10—H10B	109.5
C6—C5—C4	116.9 (4)	H10A—C10—H10B	109.5
C6—C5—C8	120.5 (4)	C8—C10—H10C	109.5
C4—C5—C8	122.5 (4)	H10A—C10—H10C	109.5
C5—C6—C1	121.6 (4)	H10B—C10—H10C	109.5
C5—C6—H2	119.2	C8—C11—H11A	109.5
C1—C6—H2	119.2	C8—C11—H11B	109.5
O1—C7—C1	123.9 (5)	H11A—C11—H11B	109.5
O1—C7—H7	118.1	C8—C11—H11C	109.5
C1—C7—H7	118.1	H11A—C11—H11C	109.5
C11—C8—C9	111.4 (6)	H11B—C11—H11C	109.5
C11—C8—C10	108.3 (5)	C2—O2—H2A	109.5
			
C6—C1—C2—O2	178.4 (4)	C4—C5—C6—C1	0.1 (6)
C7—C1—C2—O2	−1.7 (7)	C8—C5—C6—C1	179.4 (4)
C6—C1—C2—C3	−0.8 (6)	C2—C1—C6—C5	0.8 (7)
C7—C1—C2—C3	179.0 (4)	C7—C1—C6—C5	−179.1 (4)
O2—C2—C3—C4	−179.2 (4)	C6—C1—C7—O1	−179.1 (5)
C1—C2—C3—C4	0.0 (6)	C2—C1—C7—O1	1.0 (8)
O2—C2—C3—Br1	0.7 (6)	C6—C5—C8—C11	−53.8 (7)
C1—C2—C3—Br1	180.0 (3)	C4—C5—C8—C11	125.4 (6)
C2—C3—C4—C5	0.9 (7)	C6—C5—C8—C9	68.3 (6)
Br1—C3—C4—C5	−179.1 (3)	C4—C5—C8—C9	−112.5 (6)
C3—C4—C5—C6	−0.9 (6)	C6—C5—C8—C10	−174.5 (5)
C3—C4—C5—C8	179.8 (4)	C4—C5—C8—C10	4.7 (7)

### Hydrogen-bond geometry (Å, °)

**Table:**

*D*—H···*A*	*D*—H	H···*A*	*D*···*A*	*D*—H···*A*
O2—H2A···O1	0.82	1.93	2.650 (6)	145
